# Tolerance induction for EGFR TKI hypersensitivity in lung cancer: Case report and review

**DOI:** 10.1016/j.jacig.2026.100706

**Published:** 2026-04-15

**Authors:** Mizuha Haraguchi Hashiguchi, Takeya Adachi, Masato Asaoka, Tamaki Ito, Sachiko Kaburagi, Junko Kagyo, Utako Okata-Karigane, Chiaki Takahashi, Umi Tahara, Suzuyuki Yoneda, Katsunori Masaki, Koichi Fukunaga

**Affiliations:** a Keiyu Hospital, Kanagawa, Japan; b Allergy Center, Keio University Hospital, Tokyo, Japan; c Department of Dermatology, Keio University School of Medicine, Tokyo, Japan; d Department of Pediatrics, Keio University School of Medicine, Tokyo, Japan; e Division of Pulmonary Medicine, Department of Medicine, Keio University School of Medicine, Tokyo, Japan

**Keywords:** Tolerance induction, desensitization, EGFR TKIs, NSCLC, hypersensitivity

## Abstract

This report highlights the successful use of oral desensitization to manage delayed-type hypersensitivity to afatinib, enabling continuation of treatment for epidermal growth factor receptor–mutated non–small cell lung cancer. In the absence of standardized protocols, it offers valuable insights for managing similar clinical challenges.

Targeted epidermal growth factor receptor tyrosine kinase inhibitors (EGFR TKIs) have considerably improved outcomes in patients with EGFR-mutated non–small cell lung cancer (NSCLC) compared with conventional chemotherapy.^1^ However, these agents frequently cause adverse effects—primarily cutaneous (acneiform rash, xerosis, paronychia) and gastrointestinal (diarrhea)—reflecting on-target toxicity.^2^ Immunologically mediated hypersensitivity reactions, although uncommon, have also been documented. These include immediate-type reactions, such as urticaria (likely IgE-mediated), and delayed-type reactions, such as Stevens-Johnson syndrome (SJS)/toxic epidermal necrolysis and drug-induced hypersensitivity syndrome/drug reaction with eosinophilia and systemic symptoms, which are predominantly T-cell–mediated.^1^^,^^3^^,^^4^ When an EGFR TKI must be discontinued because of hypersensitivity and no equally effective alternative treatment exists, tolerance induction (for delayed reactions) or drug desensitization (for immediate reactions) may be considered to induce unresponsiveness and allow continuation of critical therapy.^5^ To date, however, experience with EGFR TKI readministration protocols remains limited. Here, we present what, to our knowledge, is the first reported case of delayed-type hypersensitivity to afatinib that was successfully managed through oral tolerance induction and review the relevant literature.

A 48-year-old man with stage IV NSCLC harboring uncommon EGFR mutations (G719S/R776H) initially received afatinib (an EGFR TKI) at a dose of 40 mg daily. Approximately 10 days after initiation of afatinib, he developed an acneiform skin rash (papulopustular eruption) and oral mucositis consistent with EGFR TKI toxicity, necessitating a dose reduction to 30 mg daily and subsequent discontinuation (Fig 1, *A*). He was then transitioned to platinum-based chemotherapy followed by long-term treatment with osimertinib (EGFR TKI), maintaining disease stability for approximately 6 years. After eventual disease progression, afatinib was reinitiated at a reduced dose (20 mg per day). Within 1 hour of rechallenge, the patient developed nausea and vomiting. On the following day, he developed high fever, and laboratory testing revealed acute kidney injury. His serum creatinine level rose to 3.64 mg/dL from a baseline of 1.67 mg/dL per day. Urinalysis revealed an increase in urinary occult blood from 1+ to 3+, in addition to the patient’s preexisting 1+ proteinuria, which improved rapidly following drug discontinuation.

**Fig 1:**
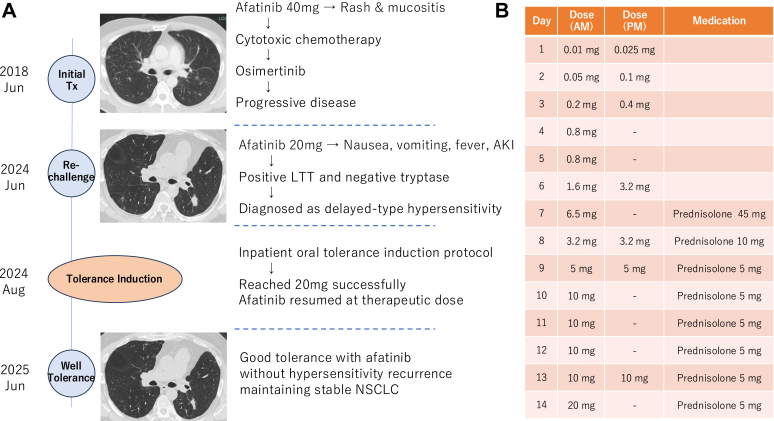
Case timeline and tolerance induction therapy. **A,** The patient discontinued initial treatment with afatinib because of rash/mucositis, received chemotherapy and long-term osimertinib (approximately 6 years), and then developed delayed-type hypersensitivity (fever, gastrointestinal symptoms, acute kidney injury [AKI]), a positive lymphocyte transformation test (LTT) result, and a negative result of testing for tryptase on afatinib rechallenge. **B,** Inpatient stepwise oral suspension (doses of 0.01 and 0.025 mg on day 1 were followed by daily dose doubling, with a dose of 20 mg reached by day 14). Mild nausea required brief prednisolone administration. Afatinib, 20 mg, was continued for more than 10 months without recurrence; the patient’s disease remained stable.

Given the timing, both immediate- and delayed-type hypersensitivity were initially considered. A negative serum tryptase test result combined with a positive result of a lymphocyte transformation test to afatinib (stimulation index 2.02; cutoff > 1.80), supported a diagnosis of delayed-type hypersensitivity. Skin testing was not performed on account of the lack of standardized reagents. Lacking alternative targeted therapies for the patient’s uncommon EGFR mutations, we planned afatinib tolerance induction and obtained informed consent. A supervised oral tolerance induction protocol using a diluted afatinib suspension allowed precise dose adjustments. On day 1, test doses of 0.01 mg and 0.025 mg were administered under close monitoring, followed by daily dose doubling (Fig 1, *B*).^6^ The patient did not develop objective signs of hypersensitivity such as fever or rash. Mild nausea occurred at an afatinib dose of 6.5 mg, prompting temporary coadministration of prednisolone. He initially received 45 mg for 1 day, followed by 10 mg on the next day, after which afatinib was gradually tapered and successfully discontinued over approximately 4 weeks. With this adjustment and a slower dose escalation, the target dose of 20 mg was successfully reached on day 14. Following the protocol, the patient continued taking afatinib at a dose of 20 mg daily, with good tolerance and no recurrence of hypersensitivity for at least 10 months. His NSCLC remains radiographically stable.

Because distinguishing immediate from delayed reactions is clinically challenging and the terminology often overlaps, we reviewed 9 cases of EGFR TKI hypersensitivity managed with desensitization or tolerance induction (Table I^3^^,^^5^^,^^7^, ^8^, ^9^). Of the 9 cases, 4 involved osimertinib, 3 involved erlotinib, 1 involved alectinib, and 1 involved afatinib. Of the 9 patients, 3 experienced immediate-type reactions (urticaria or angioedema) whereas 6 developed delayed-type reactions (SJS; punctate eruption; and systemic symptoms with fever, gastrointestinal disturbance, and acute kidney injury). The immediate-type reactions occurred within 1 to 3 days of treatment initiation. In contrast, delayed-type reactions developed after 1 to 288 days. All patients underwent induction protocols starting with very low doses and gradual escalation; antihistamines or corticosteroids were used in selected cases. In contrast to the successful cases summarized in Table I, Nagase et al^9^ reported cases involving erlotinib and alectinib, in which rapid protocols over 2 or 3 days were attempted and 1 patient (a 69-year-old female with NSCLC) could not complete the treatment. This suggests that rapid protocols, although effective for IgE-mediated reactions, may be insufficient to induce T-cell tolerance in patients with delayed-type hypersensitivity.

**Table I:** Published cases of EGFR TKI hypersensitivity successfully managed with tolerance induction/desensitization

Reference no.	Patient age (y)/sex	EGFR TKI (indication)	Hypersensitivity type and onset	Protocol	Outcome
5	59/F	Osimertinib (T790M NSCLC)	Type I (urticaria); at ∼3 d	0.1 mg → 80 mg over ∼50 d (incremental increase with H1 blockade)	Resumed at 80 mg; no recurrence of reaction
7	85/F	Osimertinib (T790M NSCLC)	Type I (urticaria); at ∼48 h	0.1 mg → 40 mg over ∼1 mo (gradual escalation)	Continued osimertinib, 40 mg; no recurrence > 12 mo
8	70/F	Osimertinib (NSCLC)	Type I (angioedema + urticaria); immediate (at first dose)	Two courses: 5 mg → 80 mg over 30 d; after 4-wk drug holiday, 5 mg → 40 mg over 2 wk	Continued osimertinib (second course at reduced dose), no further reactions
3	63/M	Osimertinib (NSCLC)	Type IV (SJS); at ∼5 wk	Initial: 0.02 mg → 80 mg over 25 d (stopped at mild rash); retrial after 5-wk pause: 40 mg → 80 mg over 5 wk	Resumed 80 mg; no SJS recurrence at 6 mo
9	75/M	Erlotinib (NSCLC)	Type IV (punctate eruption on face, oral mucosa, neck, and trunk); on d 14	0.02 mg → 100 mg over 3 d (premedication with fexofenadine, 60 mg; levocetirizine, 5 mg; and nizatidine, 150 mg)	Continued erlotinib
	70/F	Erlotinib (NSCLC)	Type IV (generalized eruption on abdomen, back, and limbs); on d 44	0.02 mg → 100 mg over 3 d (premedication with fexofenadine 60 mg, levocetirizine 5 mg, nizatidine 150 mg)	Continued erlotinib
	63/F	Erlotinib (NSCLC)	Type IV (redness on neck, trunk, and lower extremities); on d 288	0.02 mg → 75 mg over 3 d (premedication with fexofenadine, 60 mg; levocetirizine, 5 mg; and nizatidine, 150 mg)	Continued erlotinib
	43/F	Alectinib (NSCLC)	Type IV (eruption on face, neck, trunk, and limbs); on d 26	0.075 mg → 600 mg over 2 d (premedication with fexofenadine, 60 mg; levocetirizine, 5 mg; and nizatidine 150 mg)	Continued alectinib
Our case	48/M	Afatinib (NSCLC)	Type IV? (high fever + gastro-intestinal symptoms + AKI); after ∼1 d	0.01 mg →20 mg over 2 weeks (incremental increase with prednisolone from midcourse)	Continued afatinib, 20 mg; no recurrence > 10 mo

*AKI*, Acute kidney injury; *F*, female; *M*, male.

Protocols should be tailored to the immunologic mechanism.^6^ *Desensitization* is the appropriate term for preventing IgE and mast cell–mediated immediate-type reactions, during which dose escalation combined with H1-antihistamine premedication yields successful outcomes by inducing temporary mast cell nonresponsiveness.^5^ In contrast, T-cell–mediated delayed-type reactions require “tolerance induction,” which targets T-cell anergy or regulatory T-cell induction.^3^^,^^6^ In delayed-type reactions, relapse during escalation can occur; however, further slowing the escalation pace and/or adding prednisolone support, as in our case, can result in success. Although our patient atypically lacked a skin reaction following rechallenge, his positive lymphocyte transformation test result and successful tolerance induction support a T-cell–mediated mechanism. Extreme caution is required for severe reactions such as SJS/toxic epidermal necrolysis, as rechallenge can be fatal. Although theoretically unnecessary for tolerance induction, maintaining uninterrupted daily dosing may be clinically important, as one 70-year-old patient lost tolerance after a 4-week hiatus and required repeat induction.^8^

Regarding treatment selection, a prior case of afatinib-induced drug-induced hypersensitivity syndrome/drug reaction with eosinophilia and systemic symptoms managed by switching to erlotinib highlights incomplete cross-reactivity among EGFR TKIs.^1^ In our patient, however, alternative TKIs were not viable due to his mutation profile, making tolerance induction the only option. Successful management often requires protocol flexibility; for example, a 63-year-old patient with osimertinib-induced SJS required a cautious 2-step approach: an initial attempt was halted at the first sign of rash, but a second attempt after a drug-free interval succeeded.^3^ This underscores the importance of close clinical monitoring and flexibility when managing severe delayed reactions and highlights the need for multidisciplinary collaboration.

Collectively, tolerance induction and desensitization can overcome hypersensitivity reactions to EGFR TKIs, allowing patients to continue essential cancer therapies. In our patient, a carefully tailored tolerance induction protocol enabled reintroduction of afatinib without recurrence of hypersensitivity, resulting in sustained disease control. This strategy should be considered when no alternative treatments are available; it is best conducted by interdisciplinary teams to maximize patient safety and therapeutic efficacy.

## Disclosure statement

Supported in part by the Japanese Agency for Medical Research and Development (grant 25ek0410130), Japanese Society of Allergology/World Allergy Organization 2020 Memorial Research Grant Program, and SECOM Science and Technology Foundation.

Declaration of generative artificial intelligence (AI) and AI-assisted technologies in the writing process: During the preparation of this work, the authors used ChatGPT 5.0/OpenAI (Microsoft Corporation) to improve language and readability. After using this tool/service, the authors reviewed and edited the content as needed and take full responsibility for the content of the publication.

Data availability statement: The data sets generated and/or analyzed during the current study are available from the corresponding author on reasonable request.

Disclosure of potential conflict of interest: The authors declare that they have no relevant conflicts of interest.
